# A Machine-Learned Predictor of Colonic Polyps Based on Urinary Metabolomics

**DOI:** 10.1155/2013/303982

**Published:** 2013-11-07

**Authors:** Roman Eisner, Russell Greiner, Victor Tso, Haili Wang, Richard N. Fedorak

**Affiliations:** ^1^Department of Computing Science, University of Alberta, Edmonton, AB, Canada T6G 2E8; ^2^Division of Gastroenterology, Zeidler Ledcor Centre, University of Alberta, Edmonton, AB, Canada T6G 2X8; ^3^Department of Surgery, 2D2.29 WC Mackenzie Health Science Centre, University of Alberta, Edmonton, AB, Canada T6G 2R7

## Abstract

We report an automated diagnostic test that uses the NMR spectrum of a single spot urine sample to accurately distinguish patients who require a colonoscopy from those who do not. Moreover, our approach can be adjusted to tradeoff between sensitivity and specificity. We developed our system using a group of 988 patients (633 normal and 355 who required colonoscopy) who were all at average or above-average risk for developing colorectal cancer. We obtained a metabolic profile of each subject, based on the urine samples collected from these subjects, analyzed via ^1^H-NMR and quantified using targeted profiling. Each subject then underwent a colonoscopy, the gold standard to determine whether he/she actually had an adenomatous polyp, a precursor to colorectal cancer. The metabolic profiles, colonoscopy outcomes, and medical histories were then analysed using machine learning to create a classifier that could predict whether a future patient requires a colonoscopy. Our empirical studies show that this classifier has a sensitivity of 64% and a specificity of 65% and, unlike the current fecal tests, allows the administrators of the test to adjust the tradeoff between the two.

## 1. Introduction

Colorectal cancer (CRC) is a major public health concern as it is globally ranked as the third most frequent form of cancer with the age standardized incidence rate of 20.4 per 100,000 population and represents almost 8% of all deaths due to cancer [1]. It is the third leading cause of cancer-related deaths in the Western world [2]. In 2010, the National Cancer Institute estimated that there were 102,900 new cases of colon cancer, 39,670 new cases of rectal cancer, and 51,370 deaths from colon or rectal cancer in the United States [3]. In 2011, the Canadian Cancer Society estimated that 22,200 Canadians were diagnosed with CRC, which was the cause of death in 8,900 cases [4]. CRC is largely preventable through population based- and individual based-screening programs that aim to detect adenomatous polyps, the precursor to CRC. Currently, noninvasive, fecal-based testing forms the foundation of screening programs to determine which individuals should receive a colonoscopy [5], the definitive test for identifying and removing adenomatous polyps. Unfortunately, relatively few individuals complete the standard fecal-based testing, including those known to be at above-average risk for CRC [6].

Polyps in the colon are precursors to CRC, which, if detected through screening programs and removed at the precancerous stage, can help prevent CRC from developing. There are, however, several factors that limit the effectiveness of the fecal-based testing methods as screening tests. The first is low patient compliance and uptake of any fecal-based diagnostic test. Second the fecal-based diagnostic tests have low sensitivity. The guaiac-based fecal test, which tests for hemoglobin, has a sensitivity of approximately 3% for detecting any adenoma [7] and 10–30% for detecting advanced (>10 mm) adenomatous polyps [8, 9]. Newer fecal immunochemical tests, which use antibodies to globin, have reported sensitivities of 13–26% for any adenomatous polyps [7] and 20–67% for advanced adenomatous polyps [10]. Third, the interpretation of these fecal-based tests is subjective as the result is a colorimetric change, which means it can be difficult to determine whether the test is truly positive or not.

Colonoscopy is the gold standard for identifying both CRC and polyps. In an ideal world, every at-risk subject would receive a colonoscopy, since the cost of a colonoscopy (~CDN $1,000) is significantly lower for the health industry than the expected cost of treating the possible colon cancer (~CDN $20,000) [11]. However, the cost of colonoscopy and its associated morbidity and mortality precludes it as a cost effective population-based screening test. An accurate, patient compliant and inexpensive “Colonoscopy Predictor” (i.e., a test that can accurately predict whether a patient has an adenomatous polyp and so should receive a colonoscopy) would serve as the ideal population-based screening test. 

Metabolomics is a relatively new field of study, which focuses on small molecule metabolites. There are over 6,500 metabolites in the human body (and as many as 40,000) [12], whose concentrations provide a snapshot of a person's current state of health. While genomics can suggest what may develop in a particular person, a metabolomic profile provides an “up-to-the-minute” description, since these metabolite concentrations vary quickly as changes occur in the body. We used targeted-profiling metabolomics [13, 14] to obtain metabolic profiles from spot urine samples [15, 16], along with answers to clinical questions, to accurately discriminate which patients are at a greater risk of colonic polyps or colon cancer.

We hypothesized that metabolites found in urine would be indicative of colorectal cancer and its precursor adenomatous polyp and thus can be used as a population-based screening test to determine who would require a colonoscopy to remove these polyps. The aim of this study therefore was to determine whether we could learn a classifier that used the urine concentration of various metabolites, as well as answers to clinical questions, to predict whether a novel subject needed a colonoscopy (as s/he had polyps or CRC), or not. We applied machine-learning techniques to create a classifier from the data sample of historical subjects (whose “need colonoscopy” status was known) and compared its accuracy, on novel patients, with fecal-based tests.

## 2. Materials and Methods

### 2.1. Participants and Methods

#### 2.1.1. Participants

Study participants were prospectively and consecutively recruited through a population-based colon cancer screening program of asymptomatic individuals undergoing colonoscopy in Edmonton (Alberta, Canada) between April 2008 and October 2009 (SCOPE-Stop Colorectal Cancer through Prevention and Education). Participants included those at average CRC risk (50–75 years of age and no personal or first-degree family history of CRC or polyps) and at increased CRC risk (40–75 years of age with a personal or first-degree family history of CRC or polyps). Participants were excluded from the study if they were under 40 or over 75 years of age or had findings of colonic or ileal disease at the time of colonoscopy [7].

#### 2.1.2. Urine and Fecal Collections

On the day of entry into the study, participants provided informed written consent, a midstream urine sample, and a completed clinical questionnaire [7]. No dietary or activity modification was required prior to the urine collection. Urine was collected in containers that contained 6 drops of sodium azide (27.3 mg/mL; Sigma Aldrich). Samples were stored at 4°C within 4 hours and then frozen at −80°C within 24 hours. Within one week of providing the urine sample, participants provided a fecal sample for a guaiac-based fecal occult blood test and two immune-based fecal occult blood tests.

#### 2.1.3. Colonoscopy and Polyp Detection

Colonoscopy, the gold standard for identifying polyps, was performed 2 to 6 weeks after the urine and fecal collections were completed. Patients followed a standard diet and preparation procedure. Cecal intubation rate was 98% and polyp detection rate was 27%. All polyps identified were characterized according to location and size. As described in a previous publication describing the study [7], “… all 14 colonoscopists in the study were experienced endoscopists each performing a minimum of 250 procedures annually.” In all cases, the colonoscopists were blinded to the results of the fecal guaiac tests.

Colonoscopy results partition the subjects into one of four outcomes (from least severe to most severe): subjects that have (i) no abnormality (Normal); (ii) hyperplastic polyps (Hyperplastic); (iii) adenomatous polyps (Adenoma), or (iv) colorectal cancer (CRC); see Table 1. One colonoscopy patient required overnight observation in hospital for postpolypectomy syndrome and another required an appendectomy within 24 hrs of colonoscopy.

To build a predictor that can determine whether a person requires a colonoscopy, we needed a training set that has subjects who have been labeled either “Colonoscopy” (i.e., the subject had a polyp and in hindsight required a colonoscopy), or “Normal” (i.e., the subject did not require a colonoscopy). While hyperplastic polyps typically are not considered to be precancerous until these polyps are removed at colonoscopy and examined histologically, they cannot be distinguished from the precancerous adenomatous polyps. For this reason, we have included all polyps into the “Colonoscopy” group. As shown in Table 1, this labels 633 subjects as “Normal” and 110 + 243 + 2 = 355 as “Colonoscopy.”

#### 2.1.4. Fecal Occult Blood Analysis

As part of the SCOPE program, participants' fecal samples, collected 2 to 6 weeks prior to colonoscopy, underwent fecal occult blood testing using 3 commercially available tests. The Hemoccult II Beckman Coulter Canada Inc. test (nonrehydrated) was considered positive if at least 1 test window displayed a blue color within 60 s of developer. The Hemoccult ICT Beckman Coulter Inc., USA test was considered positive if a pink line appeared in the test area within 5 min of buffer. The MagStream HemSp/HT, Fujirebio Diagnostics Inc., USA test was considered positive if finding a level of 67 *μ*g Hemoglobin/g stool or above. Testing was completed by a licensed technologist at DynaLIFE DX.

#### 2.1.5. Urine Metabolomic Analysis

Urine samples, collected approximately 1 week prior to the fecal samples collection, underwent metabolomic analysis to obtain a metabolite profile.

*NMR Acquisition*. Spectra were collected using a 600 MHz NMR spectrometer (Oxford Instruments, Oxfordshire, UK) with a VNMRS two-channel console (Varian Inc., Palo Alto, CA, USA) running VNMRJ software version 2.2C on a RHEL 4 (Red Hat) host computer. The spectrometer was equipped with an HX probe with *Z*-axis gradients. The first increment of a 2D-^1^H, ^1^H-NOESY pulse sequence was utilized for the acquisition of ^1^H-NMR data and for suppressing the solvent signal. Experiments used a 100 ms mixing time along with a 990 ms presaturation (~80 Hz gammaB_1_). Spectra were collected at 25°C, with a total of 32 scans over a period of 3.5 min.

*Metabolite Quantification*. Once the spectra were acquired, quantification of 72 metabolites was completed using the targeted profiling technique of Chenomx NMRSuite v7.0 (Chenomx, Inc., Edmonton, Canada). The samples were analyzed by Chenomx as a paid service. The quantification process was completed at Chenomx by one individual and verified by a second individual blinded to the initial results. The staff at Chenomx were blinded to the results of colonoscopy and the answers to the clinical questions. From each patient profile, we excluded DSS (internal standard), ibuprofen and salicylurate (drug metabolites), and 9 other metabolites (3-hydroxybutyrate, adipate, benzoate, ethanol, galactose, glycerol, guanidoacetate, trimethylamine, urea, and beta-alanine) that were considered unreliably measured as they were difficult to profile due to both noise and additional peaks that can lead to an effect known as “tenting” (i.e., some peaks overlap with others, forming a large single peak that is actually comprised of two or more peaks). This can make quantification of a single peak much more difficult. At this point, we were left with 59 quantified metabolites.

Whenever a metabolite was not observed (perhaps because its concentrations fell below the limit of detection for NMR), we recorded its value as half of the minimum measured value for that metabolite in our sample [17]. Overall, 26% of the values in our metabolite data matrix (988 samples × 59 metabolites) had this issue. The metabolites that had the most missing values were later removed (see Section 3.1.2).

#### 2.1.6. Clinical Questionnaire

In addition to the 59 quantified metabolites for each subject, we also collected his/her answers to 4 questions thought to be relevant to CRC susceptibility: age, gender, smoking, and symptoms of gastrointestinal (GI) bleeding.  Table 2 shows the distribution of the answers to these questions in our data.

### 2.2. Statistical Methods

#### 2.2.1. Data Preprocessing

As our learning algorithms process numeric values, we had to first convert each clinical feature to numeric values. For age, no conversion was needed since the parameter was already numeric. Gender and GI bleeding data were converted to binary features. For smoking, we replaced “yes” and “no” with 1 and 0, respectively, and ex-smoking with 0.5 since this response intuitively lies somewhere between yes and no. We replaced missing values for the clinical features with the arithmetic mean of the feature over all subjects (both case and control).

The measured concentration value for each metabolite in a urine sample depends on the actual concentration of that metabolite in the subject's body, as well as the dilution factor present in the urine sample. To address this dilution effect, we considered several forms of normalization, shown in Table 3. We found, however, that some standard techniques—creatinine normalization [18], probability quotient normalization [19], vector length normalization [19], and total metabolite normalization [20, 21]—reduced the accuracy of our classifiers. Similar to results in [15], we found that log-transforming the metabolite concentrations was the only transformation that improved prediction accuracy (Section 3.1.2). We therefore used the natural logarithm, which transforms the metabolite concentration distributions (across subjects) to appear more Gaussian.

As we were not normalizing each sample, the concentration values reported for the metabolites might not be accurate. For our goal—of building an effective predictive classifier—the accuracy of specific metabolite concentrations is not critical; it is sufficient that the values be consistent. For example, a classifier could still work correctly even if the concentration value for every metabolite was twice the correct value. Our results, below, show that our values are sufficiently consistent.

#### 2.2.2. Learning a Classifier

Many metabolomic studies are univariate association studies [22]—that is, they use a historical labeled sample to try to identify the metabolites that are individually most related to the condition of study (here, “Normal” versus “Colonoscopy”). These studies help to identify candidate metabolites to further investigate in the wet lab. Although related [23], our goal is slightly different: we used the urine samples to build a classifier that would predict whether a person has a polyp and thus should undergo a colonoscopy. The distinction is important because our end goal is to produce the most accurate classifier possible based upon the metabolomic profiles of urine.

A classifier is simply a mathematical formula, *f*(**x**), that produces a predicted label $\hat{y}$, given a description **x** = [*x*_1_,…, *x*_*d*_] ∈ *ℜ* ^*d*^ of a subject, which is a vector of real values, corresponding to the concentrations of the list of metabolites, as well as the answers to the clinical questions (see description above). Classifiers that use linear equations, such as linear Support Vector Machines (SVM) (and many others), seek the parameters **w** = [*w*_1_,…, *w*_*d*_] ∈ *ℜ* ^*d*^ (a vector) and *b* ∈ *ℜ* (a constant), such that the formula

(1) $$\begin{array}{c} f_{w,b}\left(x\right)=\sum_{i=1}^{d}w_{i}\times x_{i}+b \end{array}$$

maximizes some optimization (this depends on the machine learning algorithm employed). After learning these (**w**, *b*) parameters (from the set of historical subjects), we can then use the associated classifier *f*_*w*,*b*_ to make a prediction for any new subject **x**. First, evaluate *f*_*w*,*b*_(**x**), using (1). If the resulting value is above some threshold *τ* (we initially set *τ* = 0), we predict “Colonoscopy,” otherwise we predict “Normal”:

(2) $$\begin{array}{c} {\text{Decision}}_{\tau }\left(x\right)=\{\begin{array}{cc} \text{if}\;f_{w,b}\left(x\right)\geq \tau & “\text{Colonoscopy"}\\ \text{if}\;f_{w,b}\left(x\right)<\tau & “\text{Normal."} \end{array} \end{array}$$

We discuss how we evaluate the performance of our classifiers in Section 2.3. 

#### 2.2.3. Software

All code was written in R (version 2.15.1). For SVM [24] we used the e1071 (version 1.6) R library. For naïve Bayes, we used the RWeka (version 0.4-12) library for R, which is an interface to the WEKA software [25]. For PLS-DA, KNN, and RBF SVM we used the caret R library (version 5.15-023). For LASSO [26], we used the glmnet R library (version 1.8). For C4.5 we used the C5.0 R library (version 0.1.0-15).

Some of these approaches require hyperparameters to be optimized during the training of the classifier. For SVM (RBF kernel) and KNN we used the built-in parameter tuning functionality in the caret library, with the tunelength parameter set to 10. For SVM (linear) we set the cost to the default of 1, as tuning did not help here. For PLS-DA, we used the first 2 components. For Random Forest, we used the default settings. For LASSO, we used the built-in cross-validation functionality in the glmnet package for finding the optimal lambda parameter. Finally, C4.5 and naïve Bayes do not require optimization of hyperparameters. 

### 2.3. Evaluation

Learning a classifier requires labeled training data, which describes a set of subjects using, for each subject, the feature values *x*_*i*_, along with a label *y*_*i*_ (in our case, “Colonoscopy” or “Normal”). This data is used by a machine learning algorithm (we consider several, shown in Table 4) to build a classifier. Since we are most interested in how well our classifiers can predict labels for new, unlabeled instances, we follow the standard machine learning methodology [27] of cross-validation: we first use all of the training data to produce a classifier. Then, to estimate the quality of this learned classifier, we split the data set into *k* folds (partitions). Next, each fold is used to evaluate the quality of a classifier built using the remaining data as used as a training set. Finally, we estimate the quality of the classifier built using all of the data as the mean (and standard deviation) of the qualities over these *k* classifiers.

*Sensitivity* quantifies the classifier's ability to identify positive (“Colonoscopy”) results: how often our classifier predicts “Colonoscopy,” of the cases that truly need a colonoscopy. Thus, if we use a classifier with high sensitivity, we will reliably perform colonoscopies on many subjects that need to undergo colonoscopy; this can be cost-effective, as it will lead to timely detection and hence timely treatment. However, the classifier might also incorrectly identify subjects that do not need this test, thus wasting valuable resources performing unnecessary colonoscopies.

*Specificity* deals with this other direction, by quantifying the classifier's ability to identify negative (Normal) results: how often our classifier predicts Normal, of the cases that were Normal. High specificity also saves cost since it means performing fewer unnecessary colonoscopies. However, a higher specificity could be coupled with low sensitivity, which would mean not performing the needed colonoscopies and so not detecting colorectal cancer precursors early enough.

We of course want a classifier whose sensitivity and specificity are both 100%—meaning it will give a colonoscopy to everyone who needs one and only to those people. However, this is often not possible. The threshold parameter *τ* (see (2)) allows us to trade-off sensitivity for specificity. That is, for fixed (**w**, *b*) parameters, each value of the threshold *τ* defines a classifier; we can therefore consider a whole range of different classifiers, by adjusting this *τ* value. A smaller *τ* value means more subjects will be predicted “Colonoscopy,” which leads to a large sensitivity. (As *τ* → −*∞*, we will predict that everyone should get a colonoscopy, which guarantees the sensitivity will be 1—its largest value). However, small *τ* can also reduce the specificity, as fewer of the Normals will be labeled as Normal. Analogously, specificity will rise, and sensitivity will drop with increasing *τ*. Hence, each value of *τ* leads to a (Sensitivity[*τ*], Specificity[*τ*]) pair of values.

**Figure 1:**
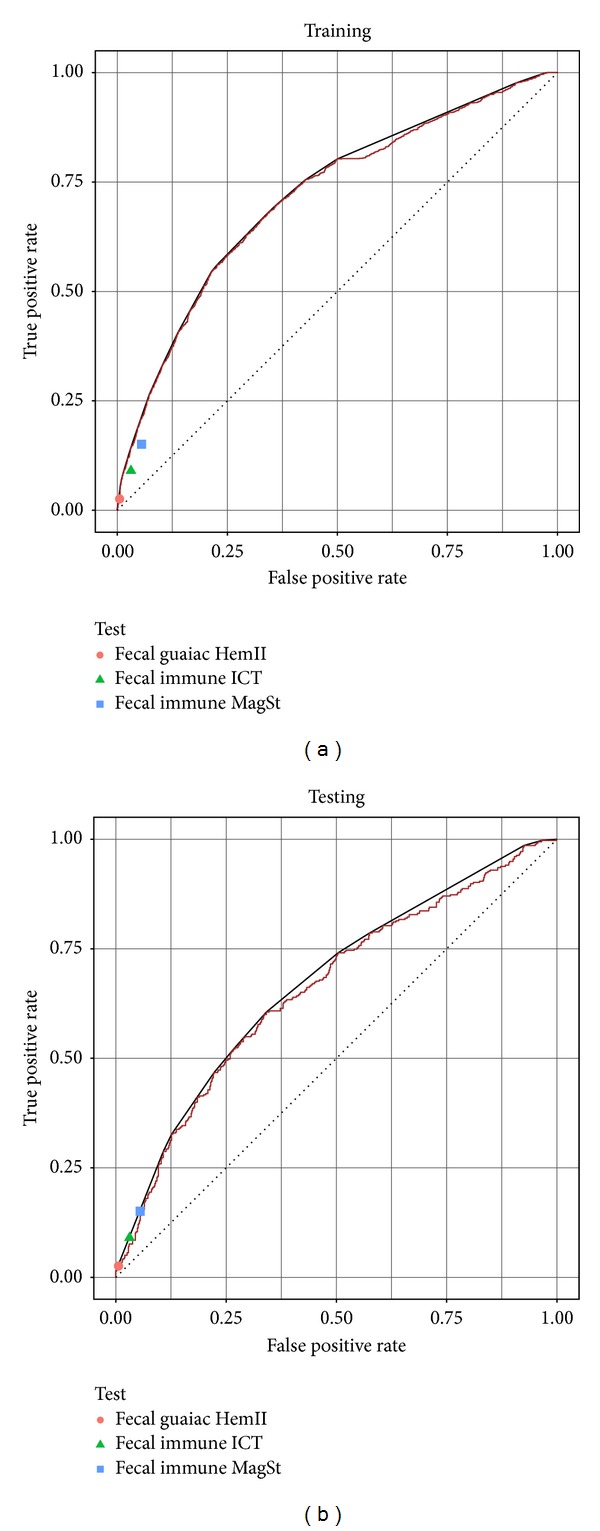
Performance of linear SVM classifier using all metabolites and clinical features. ROC curve and convex hull showing tradeoff between true positive rate (sensitivity) and false positive rate (1-specificity) for performance: (a) on the training data (resubstitution error) and (b) the testing (evaluation) data, both during cross-validation. The overall performance of the 3 tested fecal tests is also shown.

If we knew the desired (specificity, sensitivity) trade-off, we could set *τ* to optimize this—for example, if we wanted at least some specificity, we could then set *τ* to match this and then read off the sensitivity. Of course, this assumes that we know the trade-offs. If not, we could produce the ROC curves—a 2D graph, whose points correspond to (Sensitivity[*τ*], 1-Specificity[*τ*]) for various *τ* values. Note the perfect classifier corresponds to (1, 0)—the upper left point (see Figure 1). We often use the “Area under the ROC curve” (AUC) as a way to evaluate a predictor; notice this value is at most 1 (if it includes the (1, 0) point), and it should be at least 0.5 (if that line is the diagonal line from (0, 0) to (1, 1)—corresponding to randomly guessing). This AUC is an overall measure of how much we can achieve over all possible values of *τ*. (i.e, while sensitivity and specificity are measures of performance for a specific threshold *τ*, the AUC is a measure of performance over all possible *τ*'s).

## 3. Results

### 3.1. Prediction Accuracy

#### 3.1.1. Fecal-Based Tests

Fecal tests have high specificity but low sensitivity for detection of polyps.  Table 7 shows the results of running the 3 fecal tests on our Colonoscopy versus Normal data. In a clinical setting of population-based screening, a high sensitivity (i.e., we want to give colonoscopies to many of those that need them) is preferred, while maintaining a high specificity (not giving colonoscopies to subjects who do not need them). This motivates our goal of creating a classifier that can meet both requirements.

#### 3.1.2. Urine-Based Test

In our study, some of the metabolites were often below the level of detection either due to noise in the spectrum or to the detection limits of the instruments. We removed such metabolites and used only metabolites that were above the level of detection in at least 80% of the samples. This further eliminated 32 of our remaining 59 metabolites, leaving 27 metabolites. Our learning algorithm used only these, as well as the answers to the four clinical questions.

As a first test, we learned a linear SVM. Using 5-fold cross-validation, we estimate this classifier performs with an average sensitivity of 32.5% and a specificity of 90.0% when using *τ* = 0. The performance of this approach is superficially similar to the fecal-based tests: low sensitivity and high specificity. We can form the ROC curves, shown in Figure 1, by plotting the (Sensitivity[*τ*], 1-Specificity[*τ*]) points for various *τ* values. For ease of visualization, we show an aggregate ROC curve, rather than all 5 (one for each cross-validation fold).  Figure 1(a) shows the performance of the trained classifier on the training data (resubstitution error) during cross-validation and Figure 1(b), the performance on the evaluation data during cross-validation. As the ROC curves for the training set and the evaluation set have similar shapes, we can use the performance on the training data to determine our preferred tradeoff between sensitivity and specificity; and feel confident that this will generalize to future subjects. We show the convex hull of each ROC curve [28], which is the maximum achievable predictive performance we can achieve with these classifiers, if we use them stochastically [29].

For population-based screening tests, it is sometimes desirable to have a test with equal sensitivity and specificity. This corresponds to a point on the ROC curve where the two measures are equal. During the training process, we find the threshold *τ* where sensitivity is approximately equal to specificity. By doing this we achieve an average sensitivity of 67% and a specificity of 66% on the training data (using an average *τ* = −0.691). Using these *τ* thresholds on the test sets, produces an average sensitivity of 62% and specificity of 63%.

We considered a range of machine-learning techniques. Since the desired sensitivity and specificity cutoff may be different based on where the test is being used (e.g., based on the threshold for false positive and negative testing), we compared the tests using the area under the ROC curves; see Table 4. We also used SVM modified with a class-specific cost for misclassification. Since the class distribution is 422 Normal and 236 Colonoscopy, we used a cost of 1.0 for mispredicting Colonoscopy for a Normal subject and a cost of (422/236) = 1.79 for mispredicting Normal for a Colonoscopy subject.

The AUC of the various machine-learning algorithms were very similar, although the LASSO method outperformed others in learning a classifier from the full data set. We thus proceeded with further experiments using the LASSO algorithm.

Table 4 presents the results of using 27 metabolites and 4 clinical features with various machine-learning algorithms. Since LASSO performed the best, we also show the effect of using various transformations to the metabolite data (see Section 2.2.2) using LASSO. No transformations were done to the clinical data. As noted above, we achieved the highest performance when using just the log transformation.

### 3.2. Feature Selection

As noted above, we obtained the metabolite concentrations by manually fitting each subject's NMR spectrum. We can therefore reduce the cost of our classification system by reducing the number of metabolites used in our prediction formula. Furthermore, a predictor that uses fewer metabolites could perform better than one using all of the metabolites, since extra metabolites may introduce noise to the training algorithm and cause overfitting [30]. While the LASSO learning algorithm takes as input a database that includes values for all of the features, it is designed to produce a classifier that uses only a subset of those features [26]. There are also “feature selection” techniques that can reduce the set of features presented to the learner. This typically reduces the chance of overfitting and means that the resulting classifier will involve fewer features.  Table 5 shows the performance of our classifier when presented with a subset of the metabolite set (all clinical features are used in each experiment).

We considered using just the top 10 features, where the ranking is based on the correlation coefficient, minimum redundancy maximum relevance [31] (which has been applied to other human studies [32]), mutual information (which has been used successfully in mRNA research [33]), using the top SVM weights, and recursive feature elimination via SVM [34]. With each of these approaches, we sorted our metabolites from most to least important then trained a classifier with the 10 most important features. This feature selection was done infold, to avoid producing results that are unfairly optimistic.  Table 5 shows that we can achieve similar performance (to using all metabolites) by using only the 10 features with the highest correlation score, or the highest mutual information. We chose to use the correlation approach since the standard deviation of AUC was lower.

**Figure 2:**
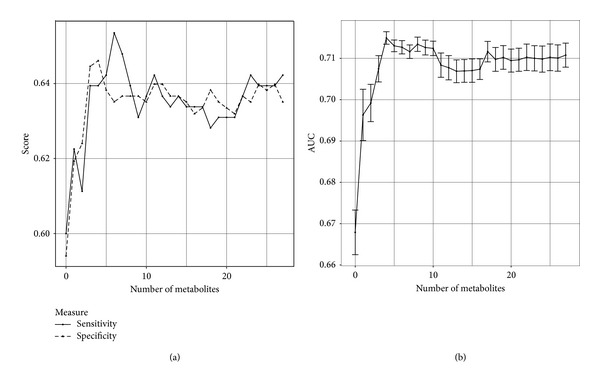
Performance of LASSO with a varying number of metabolites. Two graphs showing performance for LASSO as we vary the number of metabolites used in addition to the clinical features. (a) shows sensitivity and specificity at the selected threshold point, and (b) shows AUC for the entire ROC curve along with standard error. Note that the graphs do not show the full range [0, 1] of the *y*-axis.

We then experimented towards identifying the optimal number of features to use. Here we trained a classifier with the *k* most important metabolites, where *k* ∈ {0,…, 27}.  Figure 2 shows the (a) sensitivity, specificity, and (b) AUC, as we varied the number of metabolites. We have included the clinical features throughout, as without them, sensitivity and specificity are lower.

The performance of the classifier varied considerably for small numbers of metabolites. At the 4 metabolites mark, however, the performance began to stabilize and reached the maximum AUC. This classifier has an AUC of 0.715, and the thresholding performance has a sensitivity of 64% and a specificity of 65%.  Table 6 shows the top 4 metabolites, based on correlation coefficient on the entire data set (988 patients).

**Figure 3:**
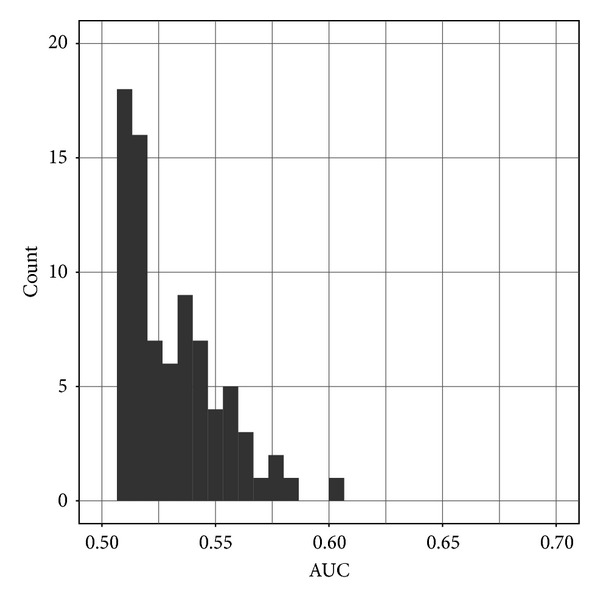
Histogram of 100 permutation test results. None of the permutation results are greater than our best classifier's performance of AUC = 0.715.

We also ran permutation tests [35] to determine whether this learning process—using LASSO on just the top 4 metabolites and all clinical features—was finding useful patterns. This involved randomizing the labels in the training set, then running our entire cross-validation process using the feature selection and LASSO processes. This was repeated 100 times. As shown in Figure 3, of these 100 permutation tests, none of the AUCs were better than of the value 0.715 based on the original unpermuted data. This supports our findings that the classifier performance is not due to random chance; that is, the chance of the null hypothesis (that we would see this 0.715 AUC performance, by chance alone) is *P* < 0.01.

## 4. Discussion

### 4.1. Predictor

Our predictor, based on urine metabolic profiles, identifies which subjects are likely to have a polyp and thus should receive a colonoscopy, with an adjustable tradeoff between sensitivity and specificity. The current standard fecal-based tests (Table 7) do not have an adjustable sensitivity or specificity threshold. Note that these tests were designed to detect colon cancer and not all polyps. However, these tests are currently used to screen for colonoscopies, and we believe that all patients with polyps should be receiving a colonoscopy.

**Figure 4:**
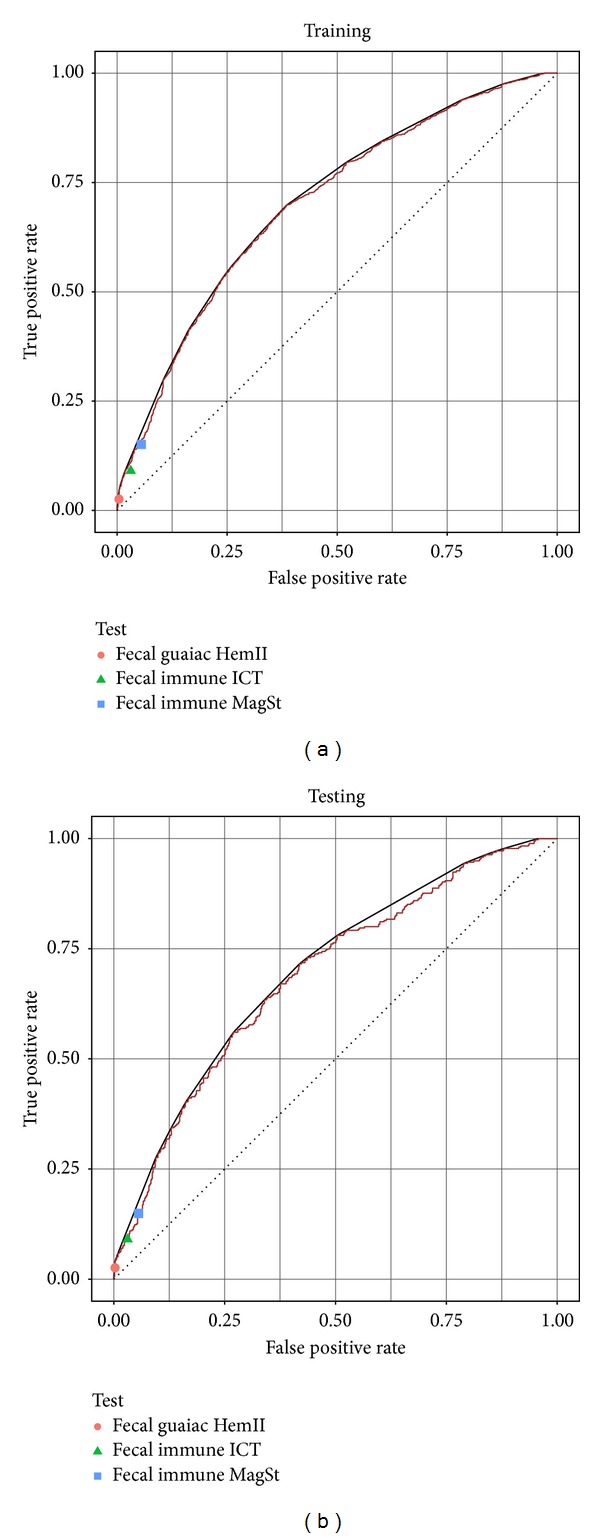
Performance of LASSO classifier using 4 metabolites and 4 clinical features. ROC curve and convex hull for our final “4 metabolite + survey questions” predictor, on the (a) training data (resubstitution error) and (b) testing (evaluation) data, both during cross-validation. The overall performance of the 3 tested fecal tests is also shown.

Figure 4 shows how well these three fecal-based tests compared to our predictor's performance in cross-validation. Since none of the fecal tests have an adjustable threshold, each corresponds to a point in the ROC space. All three fecal tests lie below our urine-based predictor's ROC curve, which indicates that on this data set, our predictor always outperforms the fecal tests. This curve shows that it is never better to use the fecal blood tests.

Figure 4(a) (as well as Figure 1) also shows the convex hull of the ROC curve, which is a series of lines connecting the outermost points of the ROC curve. The convex hull is the maximum realizable potential performance of this classifier [29], which can be attained if we stochastically combine classifiers. For example, if we want to use a point on the convex hull halfway between two thresholds (*τ*_1_ and *τ*_2_) on the ROC curve, 50% of the time we randomly choose threshold *τ*_1_, and 50% of the time we randomly choose threshold *τ*_2_). Although this may increase overall performance, clinicians or patients may not accept this, since many clinicians would not use a predictor that uses random chance.

Several previous metabolomic studies have examined patients who have colorectal cancer [36–43], adenomatous polyps [44], or various stages of colorectal cancer [45]. Our study is unique in three ways: first, we are examining a single population of patients. This differs from many studies that involve two populations—for example, CRCs from one population (say, from one geographic region) versus controls from another (different region). In those situations, the researcher cannot be sure that the observed differences are associated with the case-control differences as opposed to the population differences. In our study, all subjects were from the same population—they are all eligible subjects that appeared in our clinic between certain dates—so we expected all noncolonoscopy factors to be randomized naturally. Second, we were running a predictive study to develop a classifier that could use many features (metabolites and clinical questionnaire responses) to distinguish cases versus controls; this differs from “associative studies” that instead try to identify features (such as specific metabolites) that are individually highly associated with the case-control difference; see Section 2.2.2. Third, we explicitly sought a screening test to determine which subjects should receive a colonoscopy, which means distinguishing controls versus all polyps and colorectal cancer. This differs with other studies that try to distinguish control versus just CRC, or versus just adenomatous polyps. 

### 4.2. Features

Table 6 summarizes important features for our predictor, which is made up of 4 metabolite concentrations, and the answers to 4 clinical questions. Methanol is produced in the anaerobic metabolism of many bacteria [12]. Consequently, this breakdown can be seen in the urinary excretion. Miyagi et al. [46] has previously associated tyrosine with colorectal cancer and further discusses the relationships between amino acids and cancers. Tyrosine is also present in *E. coli *[12] and has also been linked to muscle breakdown in cancer cachexia [15]. Trigonelline is a coffee alkaloid, and has previously been associated with pancreatic cancer [47]. Acetone is a breakdown product of acetoacetate through the action of gut microflora [12] and is also found in the urine during ketosis. The clinical features are important and complement the metabolite concentrations, as classifiers that use both sets of features together do better than ones that use only one set.

### 4.3. Future Directions

We applied a wide range of learning algorithms to our dataset. The observation that they all returned essentially the same accuracy supports our belief that this behaviour is probably as good as we can expect from this data. It may be possible to produce more accurate predictors with a larger data sample (i.e., more than the 988 in our study). Our predictor is also limited by the current set of metabolites; that is, it can only find patterns over this set of metabolites. We anticipate that a predictor that can use additional metabolites may well be more accurate.

We are currently in the process of examining several factors that could affect the performance of our predictor in a real world setting. First, we are examining the effect of various stages of our protocol—such as storage conditions, sample preparation, and metabolite quantification—on the stability of our predictions. We are also looking into the day-to-day biological variance of patients, which could affect our predictions on a per patient basis. 

## 5. Conclusion

Our work is the first to use urine metabolomics to predict whether a person has polyps and so should receive a colonoscopy. This paper has precisely defined the project, with respect to a well-defined clinical task, and produced a tool that has excellent performance on that task. Our predictor, which uses only 4 urine metabolite concentrations and the answers to 4 clinical questions, performs significantly better than any of the three standard fecal blood tests, with a sensitivity of 64% and a specificity of 65%. Finally, since our study used an entire sample population (including hyperplastic and colorectal cancer subjects), it has general utility across a real clinical environment.

## Figures and Tables

**Table 1:** Results of colonoscopy for subjects in our study, along with the label we give each group for the purposes of training/evaluating a classifier.

Result of colonoscopy	Total	Label
Normal	633	Normal
Hyperplastic	110	Colonoscopy
Adenoma	243	Colonoscopy
Colorectal cancer	2	Colonoscopy

**Table 2:** Clinical features used for prediction.

Label	Age	Sex	Smoker	GI Bleeding
Colonoscopy	*μ* = 58.9	F = 159	Yes = 57	Yes = 12
	*σ* = 8.2	M = 196	Ex-smoker = 10	No = 343
			No = 274	Unknown = 0
			Unknown = 14	

Normal	*μ* = 56.2	F = 364	Yes = 57	Yes = 8
	*σ* = 8.1	M = 269	Ex-smoker = 14	No = 623
			No = 540	Unknown = 2
			Unknown = 22	

**Table 3:** Performance of LASSO Classifier using various normalization and transformation methods.

Method	Average AUC	AUC standard deviation
None	0.680	0.009
Log	**0.711**	**0.013**
Creatinine + Log	0.698	0.019
Sum + Log	0.701	0.018
Vector Length + Log	0.703	0.014
PQ + Log	0.670	0.024

The bolded row shows the approach we have decided to use.

**Table 4:** Performance of various prediction algorithms across 5 folds of cross-validation.

Model	Average AUC	AUC standard deviation
Linear-SVM	0.691	0.016
RBF-SVM	0.690	0.008
Linear-SVM + Class Cost	0.695	0.014
Naïve Bayes	0.661	0.046
PLS-DA	0.660	0.029
LASSO	**0.711**	**0.013**
Random Forest	0.692	0.015
KNN	0.611	0.033
C4.5	0.629	0.027

The bolded row shows the approach we have decided to use.

**Table 5:** Feature selection methods used to select 10 metabolites, while using a LASSO classifier and all 4 clinical features.

Method	Average AUC	AUC standard deviation
Random	0.678	0.036
Correlation (Pearson)	**0.712**	**0.008**
mRMR	0.707	0.018
Mutual information	0.712	0.015
SVM weights	0.699	0.020
SVM recursive	0.697	0.016

The bolded row shows the approach we have decided to use.

**Table 6:** Correlation coefficient for top 4 metabolites and 4 clinical features, where a positive correlation coefficient shows that the metabolite was positively correlated with patients that need to receive colonoscopies.

Feature	PubChem CID	Metabolic pathways	Correlation
Methanol	887	Gut flora metabolism	−0.16
Age			0.16
Sex			0.12
Trigonelline	5570	Nicotinate and nicotinamide metabolism	0.12
Acetone	180	Degradation of ketone bodies; propanoate metabolism	−0.11
Smoker			0.11
Tyrosine	6057	Many amino acid pathways; present in gut flora	0.09
GI Bleeding			0.07

**Table 7:** Sensitivity and specificity for fecal tests for polyp detection. Some tests are labeled as N/A because the subject did not take the test.

Test		All data	
	Sensitivity	Specificity	N/A
Fecal guaiac HemII	2.6%	99.4%	22 (2.2%)
Fecal immune ICT	9.1%	96.9%	28 (2.8%)
Fecal immune MagSt	15.1%	94.5%	23 (2.3%)

## References

[1] International Agency for Research on Cancer (2008). Estimated cancer Incidence, mortality, prevalence and disability-adjusted life years (DALYs) worldwide. *GLOBOCAN 2008*.

[2] Parkin DM, Bray F, Ferlay J, Pisani P (2005). Global cancer statistics, 2002. *CA: A Cancer Journal for Clinicians*.

[3] Colon and rectal cancer. http://www.cancer.gov/cancertopics/types/colon-and-rectal.

[4] Canadian Cancer Society's Steering Committee on Cancer Statistics (2011). *Canadian Cancer Statistics*.

[5] Leddin DJ, Enns R, Hilsden R (2010). Canadian Association of Gastroenterology position statement on screening individuals at average risk for developing colorectal cancer: 2010. *Canadian Journal of Gastroenterology*.

[6] Taylor DP, Cannon-Albright LA, Sweeney C (2011). Comparison of compliance for colorectal cancer screening and surveillance by colonoscopy based on risk. *Genetics in Medicine*.

[7] Wong CKW, Fedorak RN, Prosser CI, Stewart ME, van Zanten SV, Sadowski DC (2012). The sensitivity and specificity of guaiac and immunochemical fecal occult blood tests for the detection of advanced colonic adenomas and cancer. *International Journal of Colorectal Disease*.

[8] Allison JE, Tekawa IS, Ransom LJ, Adrain AL (1996). A comparison of fecal occult-blood tests for colorectal-cancer screening. *The New England Journal of Medicine*.

[9] Imperiale TF, Ransohoff DF, Itzkowitz SH, Turnbull BA, Ross ME (2004). Fecal DNA versus fecal occult blood for colorectal-cancer screening in an average-risk population. *The New England Journal of Medicine*.

[10] Whitlock EP, Lin JS, Liles E, Beil TL, Fu R (2008). Screening for colorectal cancer: a targeted, updated systematic review for the U.S. Preventive Services Task Force. *Annals of Internal Medicine*.

[11] Maroun J, Ng E, Berthelot J-M (2003). Lifetime costs of colon and rectal cancer management in Canada. *Chronic Diseases in Canada*.

[12] Wishart DS, Jewison T, Guo AC (2013). HMDB 3.0—the human metabolome database in 2013. *Nucleic Acids Research*.

[13] Weljie AM, Newton J, Mercier P, Carlson E, Slupsky CM (2006). Targeted pofiling: quantitative analysis of ^1^H-NMR metabolomics data. *Analytical Chemistry*.

[14] Wishart DS (2008). Quantitative metabolomics using NMR. *TrAC—Trends in Analytical Chemistry*.

[15] Eisner R, Stretch C, Eastman T (2011). Learning to predict cancer-associated skeletal muscle wasting from ^1^H-NMR profiles of urinary metabolites. *Metabolomics*.

[16] Stretch C, Eastman T, Mandal R (2012). Prediction of skeletal muscle and fat mass in patients with advanced cancer using a metabolomic approach. *Journal of Nutrition*.

[17] Xia J, Psychogios N, Young N, Wishart DS (2009). MetaboAnalyst: a web server for metabolomic data analysis and interpretation. *Nucleic Acids Research*.

[18] Holmes E, Foxall PJD, Nicholson JK (1994). Automatic data reduction and pattern recognition methods for analysis of ^1^H nuclear magnetic resonance spectra of human urine from normal and pathological states. *Analytical Biochemistry*.

[19] Dieterle F, Ross A, Schlotterbeck G, Senn H (2006). Probabilistic quotient normalization as robust method to account for dilution of complex biological mixtures. Application in ^1^H-NMR metabonomics. *Analytical Chemistry*.

[20] Bollard ME, Stanley EG, Lindon JC, Nicholson JK, Holmes E (2005). NMR-based metabonomic approaches for evaluating physiological influences on biofluid composition. *NMR in Biomedicine*.

[21] Craig A, Cloarec O, Holmes E, Nicholson JK, Lindon JC (2006). Scaling and normalization effects in NMR spectroscopic metabonomic data sets. *Analytical Chemistry*.

[22] Spratlin JL, Serkova NJ, Eckhardt SG (2009). Clinical applications of metabolomics in oncology: a review. *Clinical Cancer Research*.

[23] Madsen R, Lundstedt T, Trygg J (2010). Chemometrics in metabolomics—a review in human disease diagnosis. *Analytica Chimica Acta*.

[24] Vapnik V (1995). *The Nature of Statistical Learning Theory*.

[25] Frank E, Hall M, Holmes G, Maimon O, Rokach L (2010). Weka-A machine learning workbench for data mining. *Data Mining and Knowledge Discovery Handbook*.

[26] Tibshirani R (1996). Regression shrinkage and selection via the Lasso. *Journal of the Royal Statistical Society B*.

[27] Hastie T, Tibshirani R, Friedman J (2001). *The Elements of Statistical Learning*.

[28] Provost F, Fawcett T (1997). *Analysis and Visualization of Classifier Performance: Comparison under Imprecise Class and Cost Distributions*.

[29] Scott MJJ, Niranjan M, Prager RW Realisable classifiers: improving operating performance on variable cost problems.

[30] Russell SJ, Norvig P (2002). *Artificial Intelligence: A Modern Approach*.

[31] Ding C, Peng H (2005). Minimum redundancy feature selection from microarray gene expression data. *Journal of Bioinformatics and Computational Biology*.

[32] Huang T, Wan S, Xu Z (2011). Analysis and prediction of translation rate based on sequence and functional features of the mRNA. *PLoS ONE*.

[33] Sun L, Yu Y, Huang T (2012). Associations between ionomic profile and metabolic abnormalities in human population. *PLoS ONE*.

[34] Guyon I, Weston J, Barnhill S, Vapnik V (2002). Gene selection for cancer classification using support vector machines. *Machine Learning*.

[35] Pesarin F (2001). *Multivariate Permutation Tests: With Applications in Biostatistics*.

[36] Qiu Y, Cai G, Su M (2009). Serum metabolite profiling of human colorectal cancer using GC-TOFMS and UPLC-QTOFMS. *Journal of Proteome Research*.

[37] Cheng Y, Xie G, Chen T (2012). Distinct urinary metabolic profile of human colorectal cancer. *Journal of Proteome Research*.

[38] Wang W, Feng B, Li X (2010). Urinary metabolic profiling of colorectal carcinoma based on online affinity solid phase extraction-high performance liquid chromatography and ultra performance liquid chromatography-mass spectrometry. *Molecular BioSystems*.

[39] Wang H, Schiller DE, Tso V, Slupsky C, Wong CK, Fedorak RN A novel highly sensitive test for detecting colon cancer using spot urine metabolomics. *Gastroenterology*.

[40] Qiu Y, Cai G, Su M (2010). Urinary metabonomic study on colorectal cancer. *Journal of Proteome Research*.

[41] Nishiumi S, Kobayashi T, Ikeda A (2012). A novel serum metabolomics-based diagnostic approach for colorectal cancer. *PLoS ONE*.

[42] Mal M, Koh PK, Cheah PY, Chan ECY (2012). Metabotyping of human colorectal cancer using two-dimensional gas chromatography mass spectrometry. *Analytical and Bioanalytical Chemistry*.

[43] Ma Y, Zhang P, Wang F, Liu W, Yang J, Qin H (2012). An integrated proteomics and metabolomics approach for defining oncofetal biomarkers in the colorectal cancer. *Annals of Surgery*.

[44] Wang H, Tso VK, Slupsky CM, Fedorak RN (2010). Metabolomics and detection of colorectal cancer in humans: a systematic review. *Future Oncology*.

[45] Farshidfar F, Weljie AM, Kopciuk K (2012). Serum metabolomic profile as a means to distinguish stage of colorectal cancer. *Genome Medicine*.

[46] Miyagi Y, Higashiyama M, Gochi A (2011). Plasma free amino acid profiling of five types of cancer patients and its application for early detection. *PLoS ONE*.

[47] Arlt A, Sebens S, Krebs S (2012). Inhibition of the Nrf2 transcription factor by the alkaloid trigonelline renders pancreatic cancer cells more susceptible to apoptosis through decreased proteasomal gene expression and proteasome activity. *Oncogene*.

